# A New Planar Potentiometric Sensor for In Situ Measurements

**DOI:** 10.3390/s24082492

**Published:** 2024-04-12

**Authors:** Nikola Lenar, Robert Piech, Beata Paczosa-Bator

**Affiliations:** Faculty of Materials Science and Ceramics, AGH University of Krakow, Mickiewicza 30, PL-30059 Krakow, Poland

**Keywords:** potentiometric sensor, pH measurement, potassium determination, planar construction, in situ measurement, environmental samples

## Abstract

A new construction of a potentiometric sensor was introduced for the first time. It relies on the use of two membranes instead of one, as in the well-known coated-disc electrode. For this purpose, a new electrode body was constructed, including not one, but two glassy carbon discs covered with an ion-selective membrane. This solution allows for the sensor properties to be enhanced without using additional materials (layers or additives) on the membrane. The new construction is particularly useful for in situ measurements in environmental samples. Two ion-selective polymeric membranes were used, namely H^+^ and K^+^-selective membranes, to confirm the universality of the idea. The tests conducted included chronopotentiometric tests, electrochemical impedance spectroscopy, and potentiometric measurements. The electrical and analytical parameters of the sensors were evaluated and compared for all tested electrodes to evaluate the properties of the planar electrode versus previously known constructions. Research has shown that the application of two membranes instead of one allows for the resistance of an electrode to be lowered and for the electrical capacitance to be elevated. Improving the electrical properties of an electrode resulted in the enhancement of its analytical properties. The pH measurement range of the planar electrode is 2–11, which is much wider in contrast to that of the single-membrane electrode. The linear range of the K^+^-selective planar electrode is wider than that of the coated-disc electrode and equals 10^−6^ to 10^−1^ M. The response time turned out to be a few seconds shorter, and the potential drift was smaller due to the application of an additional membrane in the electrode construction. This research creates a new opportunity to design robust potentiometric sensors, as the presented construction is universal and can be used to obtain electrodes selective to various ions.

## 1. Introduction

The history of ion-selective electrodes (ISEs) dates back to the beginning of the twentieth century, when Cremer noticed that the electrical potential of a thin glass membrane depends on the pH of the solution [1]. This finding subsequently led to the invention of the glass electrode [2]. By the 1960s, ISEs underwent a major development that laid the foundation for modern potentiometric sensors. Ever since 1961 when Pungor [3,4] presented halide-selective electrodes with heterogeneous membranes, several breakthrough inventions created a new era in potentiometric analysis, including crystalline membrane ISEs (including a fluoride electrode with an LaF crystal) [5], neutral ionophore-based liquid membrane ISEs [6,7], and charged ionophore-based liquid membrane ISEs [8]. Plasticized poly(vinyl chloride) (PVC) was constantly used as the standard matrix of the liquid membrane in this period, which still remains highly relevant today [9]. Since then, these ISEs based on solid membranes (e.g., glassy membranes and crystalline membranes) or liquid membranes (e.g., polymeric membranes) have attracted extensive research attention and experienced a golden period of development extending to today [10,11]. During this period, various construction solutions have been invented to improve both the analytical and performance properties of ion-selective electrodes.

The first alternative for the conventional construction was the coated-wire electrode, which is obtained by covering the platinum wire with ion-selective membrane. The first construction of an electrode without the inner solution was presented in the literature in the early 1970s by Cattrall et al. [12]. Subsequently, the wire was replaced with a disc electrode, and the coated-disc electrode was presented [12,13]. The idea was to eliminate the inner solution and simply apply a membrane to the electrode’s surface. This construction was associated with disadvantages caused by the limited charge transfer processes at the membrane–electrode interface, such as the poor stability of the potentiometric response and the prolonged time of response.

The efficient transduction between the ion-selective membrane and the electrode’s surface became possible when the additional layers were implemented into the sensors’ construction, and this type of ISE was named a solid-contact ISE (SC-ISE) [13].

In 1989, the new construction was presented—the double-membrane electrode—where not one, but two membranes of different kind—external and internal—were applied onto the electrode [14]. This electrode was based on an internal conductor membrane and another external electroactive membrane.

Another construction idea presented in the literature was SPEs (screen-printed electrodes). These potentiometric sensors are obtained by covering commercially available screen-printed electrodes with polymer membranes containing ionic liquids [15]. Screen-printing is especially suitable due to its miniature size, simplicity, low-cost, high reproducibility, and efficiency in large-scale production [16]. Various potentiometric sensors, including pH electrodes based on metal oxides [17,18] and zeolites [19], as well as various ion-selective electrodes based on liquid membranes [20], have been manufactured using screen printing.

One of the latest presented construction ideas is the Rotating Ring Disk Electrode (RRDE). The RRDE is very similar to an RDE, where both use a similar hydrodynamic approach. However, the main difference between an RRDE and an RDE is the addition of a second working electrode in the form of a ring around the central disk of the first working electrode. The potentiometric rotating ring disk electrode (RRDE) includes a pH-sensing ring [21].

The reason behind creating all of the new construction solutions for ISEs is that the potentiometry method is developing rapidly thanks to its great advantages over other analytical methods. The potentiometry method using ion-selective electrodes can be characterized as a reliable method with a short response time, high selectivity, and great cost-effectiveness [22,23]. The potentiometric setup is not only inexpensive and uncomplicated, but can also be miniaturized into a portable size. What should be emphasized is that potentiometry allows for the determination of an ionic form of elements, which have the highest reactivity and bioavailability and influence living organisms the most.

In this paper, we present a brand-new construction of an ion-selective electrode by applying two identical membranes on two sides of an electrode’s body. For this purpose, a new disc electrode was constructed with two glassy carbon discs placed opposite to each other. This new construction was used to produce the new potentiometric sensor named the planar sensor. This idea can be introduced into the process of conducting in situ measurements of environmental samples. The new construction is universal and can be covered with various ion-selective polymeric membranes depending on the analyzed ions. In the scope of this paper, two PCV-based membranes were tested—potassium- and hydrogen-selective membranes. The electrical and analytical properties of the new planar electrode were compared with those of the commercial glass electrode and the well-known coated-disc electrodes (with the same H^+^-selective and K^+^-selective membranes as the planar electrodes).

## 2. Materials and Methods

### 2.1. Chemicals

The chemicals used in the scope of the experiment included inorganic salts and acids for the preparation of standard solutions and membrane components.

Standard pH solutions were buffered with 10 mM of citric acid and 10 mM of boric acid titrated with sodium hydroxide or hydrochloric acid to meet the desired pH values.

Hydrogen-selective membrane components that included hydrogen ionophore V (Calix [4]-aza-crown), lipophilic salt-sodium tetrakis(4-fluorophenyl)borate dihydrate, 2-nitrophenyl octyl ether (o-NPOE), and high-molecular-weight poly(vinyl chloride) (PVC) were purchased from Sigma-Aldrich, Saint Louis, MO, USA. Tetrahydrofuran, used as a solvent, was also purchased from Sigma-Aldrich, MO, USA.

Standard K^+^ ion solutions were obtained by diluting the standard 1 M KCl solution (potassium chloride was purchased from POCH, Gliwice, Poland) with deionized water to obtain the series of solutions with decreasing concentrations (10^−^^7^ to 10^−^^1^ M KCl).

The potassium-selective membrane components included potassium ionophore I (valinomycin), lipophilic salt-potassium tetrakis(4-chlorophenyl)borate, 2-nitrophenyl octyl ether (o-NPOE), and PVC, which were all purchased from Sigma-Aldrich. For this membrane, THF was also used as the solvent.

Sodium chloride, iron (II) chloride, and iron (III) chloride were used in the form of an aqueous solution for further reversibility and redox sensitivity tests. All salts and acids were purchased from POCH, Poland.

All chemicals were of analytical grade and were used as received without any further purification.

### 2.2. Preparation of Sensors

The first step was the preparation of the membranes. For the purpose of this research, two different membranes were prepared to investigate the sensor’s performance: hydrogen-selective and potassium-selective membranes. The composition of H^+^-ISM was as follows: 0.90% (*w*/*w*) hydrogen ionophore V, 66% (*w*/*w*) o-NPOE, 32.85% (*w*/*w*) PVC, and 0.25% (*w*/*w*) sodium tetrakis(4-fluorophenyl)borate dihydrate.

The K^+^-ISM membrane included 1.10% (*w*/*w*) potassium ionophore I, 65.65% (*w*/*w*) o-NPOE, 33.00% (*w*/*w*) PVC, and 0.25% (*w*/*w*) potassium tetrakis(4-chlorophenyl)borate. All mentioned components were dissolved in tetrahydrofuran (THF).

The series of coated-disc electrodes (with one membrane) was prepared by casting the surface of glassy carbon disc electrodes with PEEK corpus (Mineral, Sadowa, Poland) with ion-selective membrane by dropping a specific amount of membrane solution in THF with the pipette. The sensor was kept at room temperature until THF evaporation. The glassy carbon disc electrode was casted twice with 30 µL of membrane solution. Five items of potassium-selective membranes and five items of hydrogen-selective coated-disc electrodes were prepared using the exact same procedure.

The preparation of the planar electrode with two ion-selective membranes began with designing the body of the electrode. First, the glassy carbon cylinder was connected to the metallic rod to ensure conductivity. The circuit thus obtained was submerged in epoxy and, after drying, the electrode was polished to receive two glassy carbon discs facing each other. The discs were polished with alumina slurries and prepared similarly to the procedure used for the single glassy carbon disc electrodes. The cleaned and dried surfaces of the glassy carbon cylinder were covered with polymeric membranes (hydrogen and potassium PVC-based membranes) using the drop casting technique. Each side of the corpus of an electrode was casted twice with 30 µL of membrane solution. Five items of a potassium-selective planar electrode and five items of hydrogen-selective planar electrodes were obtained.

**Figure 1:**
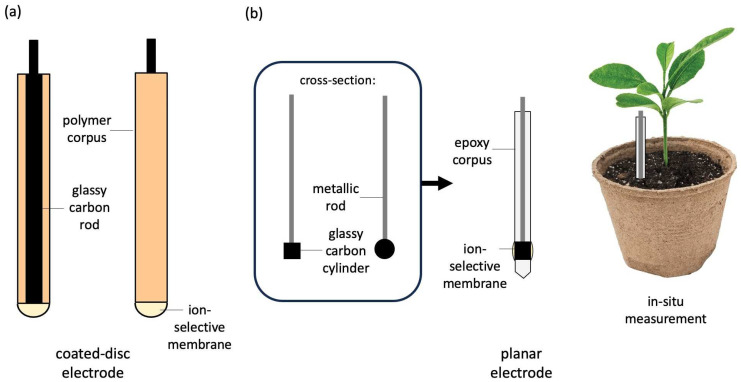
The construction of a coated-disc electrode (**a**) and planar electrode (**b**). The scheme presents the exemplary in situ measurement in the flowerpot conducted with the use of a planar sensor.

A schematic representation of the construction of both discussed electrodes, coated-disc and planar electrodes, is presented in Figure 1 for comparison.

The unique shape of the planar electrode makes it easier to perform in situ measurements in environmental samples. The placement of an ion-selective membrane in the planar construction is more convenient than the membrane position in the coated-disc electrode, which makes the new construction more resistant and durable. Both electrodes require the use of the same chemicals and components, yet the new planar construction ensures significantly improved performance parameters in comparison with the coated-disc electrode.

### 2.3. Methods

Designed sensors were tested with the use of the following electrochemical methods: potentiometry, chronopotentiometry, and electrochemical impedance spectroscopy.

The potentiometry method was applied to evaluate the analytical properties of the sensor, such as its sensitivity to hydrogen and potassium ions, repeatability, reproducibility and stability of the potentiometric response, and time of response. Measurements were made with the use of a 16-channel potentiometer (Lawson Labs, Inc., Malvern, PA, USA) with reference to the silver chloride electrode (Ag/AgCl electrode) with a 3 M KCl solution (Metrohm, Zofingen, Switzerland) and in the presence of an auxiliary electrode (platinum rod).

All five items of hydrogen-selective planar electrodes were connected to the potentiometer, experiments were carried out using the series of standard pH solutions buffered with citric and boric acid, and pH values were adjusted using hydrochloric acid and sodium hydroxide (from pH 2 to 12). The sensor with two membranes was tested together with a coated-disc electrode with one membrane and a commercial glass electrode (type 6.0150.100 by Metrohm, Switzerland) to compare its analytical properties with other well-known constructions.

Potassium selective planar electrodes (five items) were tested in reference to coated-disc electrodes with exactly the same polymeric membrane using a series of potassium chloride solutions of increasing concentration (from 10^−^^7^ to 10^−^^1^ M KCl).

Chronopotentiometric measurement was performed with the use of Autolab General Purpose Electrochemical System (AUT302N.FRA2-AUTOLAB, Metrohm Autolab, Utrecht, The Netherlands). The tested sensor was connected to the analyzer together with the reference Ag/AgCl electrode (Metrohm, Switzerland) and the auxiliary electrode (a glassy carbon rod). The measurement was performed in the standard pH 3 solution according to the following procedure: the positive sign current was forced to flow through the cell for 60 s and then switched to the negative sing current. This scheme was repeated three times to receive six steps. Chronopotentiograms were recorded using Nova 2.1 software. The electrical capacitance parameter (C) was calculated using the equation ΔEdc/Δt = I/C, where ΔEdc/Δt is the potential drift and I represents the current forced to flow through the cell.

Electrochemical impedance spectroscopy measurement was performed using the same setup. The sensor was scanned in the frequency (ƒ) range between 100 kHz and 0.01 Hz with an amplitude of 50 mV and an applied potential of 0.15 V. Recorded Nyquist plots (on which the imaginary impedance (Z^″^) is plotted as a function of the occurring impedance (Z^′^)) were interpreted using Nova 2.1 software (by Metrohm Autolab, The Netherlands), and the electrical circuit was fitted to the obtained results. The resistance parameter was obtained based on the equivalent circuit, and the electrical capacitance parameter was calculated for the lowest frequency (0.01 Hz) using the following equation: C = 1/(2πƒZ^″^).

## 3. Results

### 3.1. Ionic Response

The potentiometric response of the planar sensor was tested towards hydrogen and potassium ions. The hydrogen-selective planar electrodes were examined together with the series of coated-disc electrodes with the same polymeric hydrogen membrane and the conventional electrode, the glass electrode. The measurements were conducted through a period of five days to record the calibration curves for all tested electrodes in the standard buffer solutions of pH 2–12.

The potassium-selective planar electrode was tested in the presence of the series of coated-disc electrodes with the same polymeric potassium membrane.

**Figure 2:**
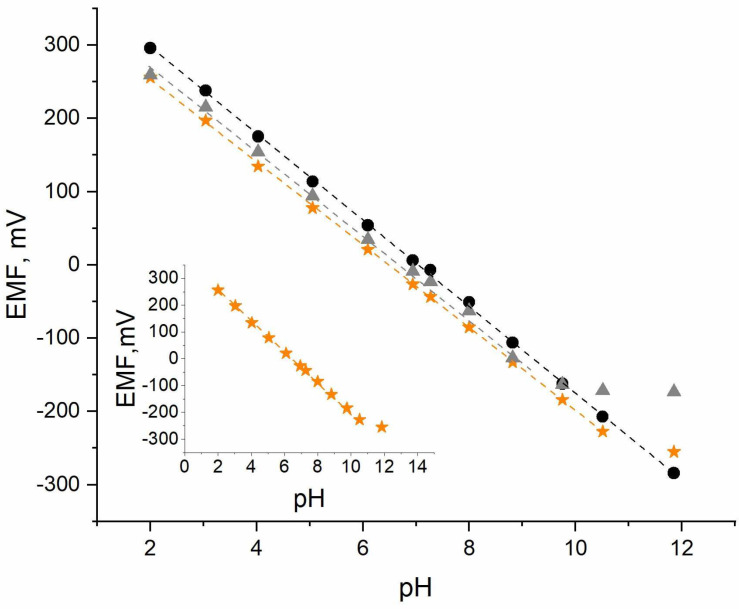
Exemplary potentiometric responses of the 🟊 planar H^+^-selective electrode, ▲ coated-disc H^+^-selective electrode, and ⏺ glass electrode towards hydrogen ions recorded in the standard pH solutions (pH 2–12) after 24 h of conditioning in the standard buffer solution with pH 3.

**Figure 3:**
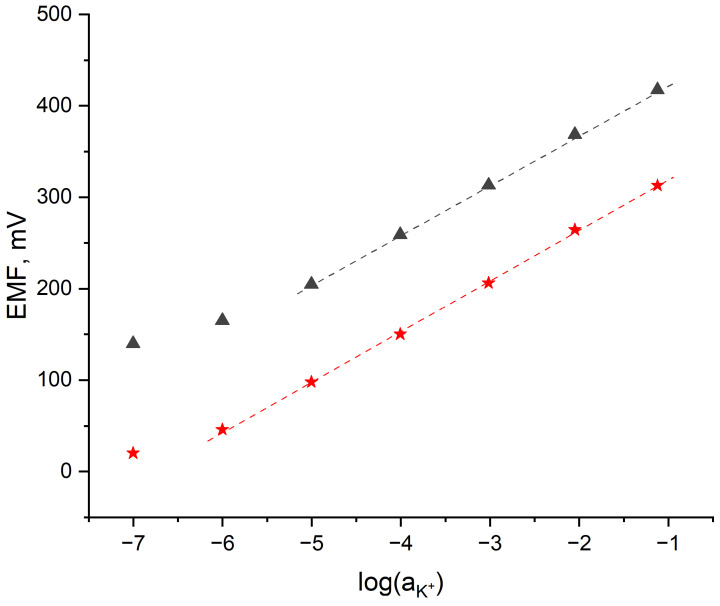
Exemplary potentiometric responses of the 🟊 planar K^+^-selective electrode and ▲ coated-disc K^+^-selective electrode towards potassium ions recorded in the standard K^+^ solutions (10^−7^ to 10^−1^ M) after 24 h of conditioning in the standard 10^−1^ KCl solution.

The exemplary potentiometric response obtained for one of the hydrogen-selective electrodes representing each group after 24 h of conditioning in the pH 3 buffer solution is presented in Figure 2, while the potentiometric response of the potassium-selective electrodes representing each group after 24 h of conditioning in the 10^−^^1^ M KCl solution is presented in Figure 3.

The analytical parameters of the planar electrodes obtained based on the measurements (during the period of 5 days) are presented in Table 1 and compared with the parameters of the glass electrode and the coated-disc electrode.

Based on the calibration curves (Figure 2 and Figure 3) and their parameters (Table 1), it can be concluded that the addition of the extra membrane allows the pH measurement range and the linear K^+^ ion range to be minimally expanded in contrast to the coated-disc construction.

In comparison to the coated-disc H^+^-selective electrode, the planar electrode can be used for pH measurements ranging from 2 to 10.3 (while the well-known construction with one membrane can be used in a narrower range from 2 to 9.3). The pH range obtained for the new planar construction is almost as wide as that for the glass electrode (2–12). A slight improvement was observed in the lowest concentration range, which was more visible for the pH electrode.

The slope of the calibration curve of the planar pH electrode is 56.6 mV/pH, which is close to the theoretical Nernstian value.

In comparison to the coated-disc K^+^-selective electrode, the planar electrode can be used for the determination of potassium ions in a wider range of concentrations, such as 10^−6^–10^−1^ M. The slope of the calibration curve of the planar K^+^ electrode is 55.2 mV/pH.

### 3.2. Repeatability of Potentiometric Response

**Figure 4:**
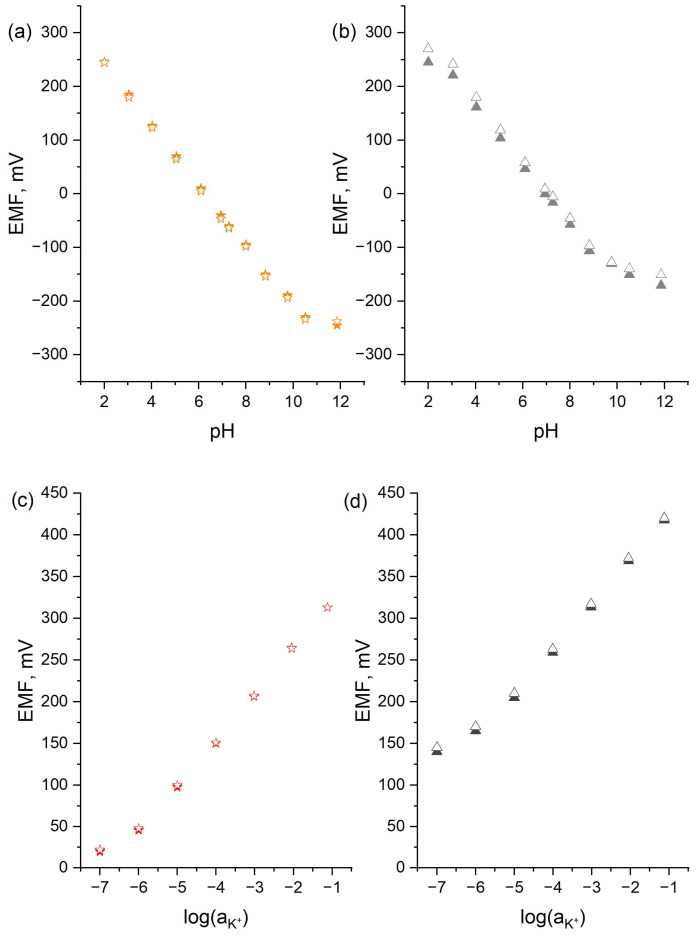
The calibration curves obtained when analyzing the pH standard solutions (2–12 and 12–2) using the 🟊 planar H^+^-selective electrode (**a**) and ▲ coated-disc H^+^-selective electrode (**b**) and the K^+^ ion standard solutions (10^−7^ to 10^−1^ M and 10^−1^ to 10^−7^ M) using the 🟊 planar K^+^-selective electrode (**c**) and ▲ coated-disc K^+^-selective electrode (**d**). The solid shapes refer to the decreasing concentration of ions, and the blank ones refer to the increasing concentration of ions.

The repeatability of the tested electrodes was evaluated on the basis of the results obtained during the potentiometric measurements conducted in a certain period. The repeatability of electrodes of different constructions can be compared on the basis of the standard deviation values in Table 1, and the results are presented in Figure 4.

Table 1 presents the analytical parameters of the tested electrodes together with the standard deviation values obtained based on the measurements conducted during the five days of electrode conditioning. The standard deviation values depict the repeatability of electrodes with the smallest values obtained for the glass electrode and the planar electrodes (both H^+^-selective and K^+^-selective). The values are much higher for the coated-disc electrodes, which display worse repeatability, compared to those of the electrode with two membranes instead of one.

Figure 4 presents the results obtained for the planar (a,c) and coated-disc (b,d) electrodes during the calibrations performed when elevating and then lowering the ion concentration (K^+^ and H^+^) of the analyzed solutions (after 120 h hours of electrode conditioning). Both curves should overlap to ensure the best repeatability. For the electrodes with two membranes, the first curve covers the second nearly perfectly, while the curves obtained for the coated-disc electrodes are divergent.

Taking into consideration all obtained results, it can be concluded that the new construction of an ion-selective electrode presented in the scope of this work gives a more repeatable response towards hydrogen and potassium ions and can be further developed to design a free-calibration sensor.

### 3.3. Reproducibility of Potentiometric Response

**Figure 5:**
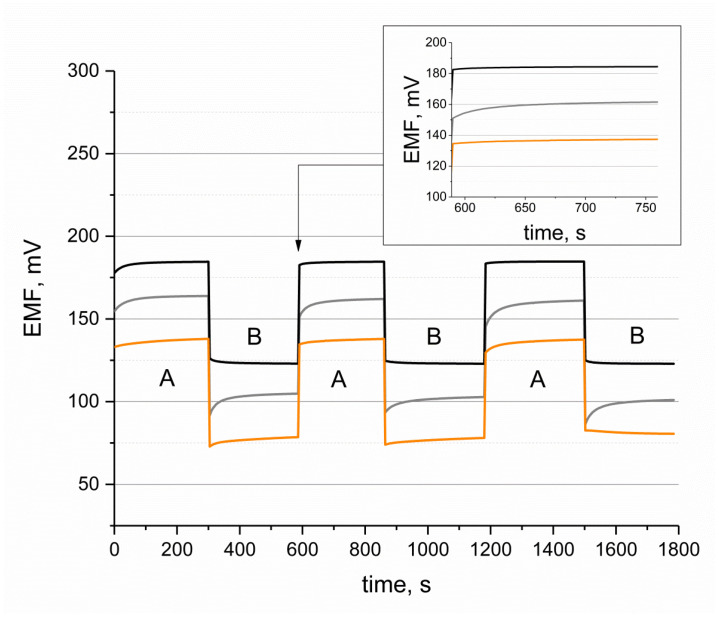
Reproducibility of planar H^+^-selective electrode (orange line), coated-disc H^+^-selective electrode (grey line), and glass electrode (black line) after contacting hydrogen ions of different concentrations recorded in standard pH solutions of pH 4 (A) and 5 (B). Inset: time of response of H^+^-selective electrodes.

The reproducibility of the potentiometric response was tested for both the hydrogen- and potassium-selective planar electrodes. The results presented in this paper refer to the pH planar sensor. For hydrogen-selective electrodes, the tests were carried out by changing the pH of the standard buffer solution (Figure 5). The test was performed during potentiometric measurements using the planar electrode, the glass electrode, and the coated-disc electrode.

Figure 5 presents the stabilization of potential over time while changing the pH of the standard buffer solution from pH 4 to pH 5. This procedure was repeated three times. During this measurement, the reversibility of the potential of the planar electrode was almost as good as that of the glass electrode. In the case of the coated-disc electrode, the stabilization of the potentiometric response was much slower, and significant potential drift was observed when changing the pH of the buffer solution.

### 3.4. Response Time

The time of the potentiometric response was calculated based on the results presented in Figure 5 (see Figure 5 inset). According to the IUPAC recommendations, the time of response is the time needed to reach 95% of the equilibrium potential [24]. For a coated-disc electrode, the calculated time of response is 13 s. For the glass and planar H^+^-selective electrodes, the potential stabilizes almost immediately, and the 95% equilibrium potential is reached in less than a few seconds.

An analogous test was performed for the K^+^-selective planar electrode. The estimated response time is also less than a few seconds.

It can be concluded that planar electrodes exhibit a remarkably short response time.

### 3.5. Potential Stability

Potential stability is described by the potential drift parameter calculated for each electrode on the basis of the results obtained during the long-time measurement in the standard ion solution. For the hydrogen-selective electrodes, the test was carried out in the standard pH solution of pH 3, while for the potassium-selective electrodes, the test was carried out in the standard K^+^ solution of a 10^−1^ M concentration.

For the planar H^+^-selective electrode, the potential drift is 0.12 mV/h, which is significantly lower compared to the one calculated for the coated-disc electrode (0.67 mV/h). For the commercial glass electrode, the potential drift is 0.019 mV/h.

For the planar K^+^-selective electrode, the stability of the potentiometric response given by the potential drift parameter (0.092 mV/h) is improved, contrary to the stability of the coated-disc electrode (0.97 mV/h).

The results prove that the stability of the potentiometric response of ion-selective electrodes can be improved using a presented planar construction. The addition of another opposite layer of membrane in the electrode construction allowed for the stabilization of the potentiometric response of H^+^ and K^+^-selective ISEs.

The lifetimes of the coated-disc electrode and planar sensor were similar. In both cases, a change in the potentiometric response was observed during use, which is typical for this type of construction, i.e., without the use of hydrophobic intermediate layers. However, in the case of coated-disc electrodes, we often observed the membrane detaching from the substrate. And this happened very often in the real sample tests. Such a problem was significantly reduced in the planar construction by using a different type of substrate and a symmetrical location for the ion-selective membranes.

### 3.6. Redox Sensitivity Test

**Figure 6:**
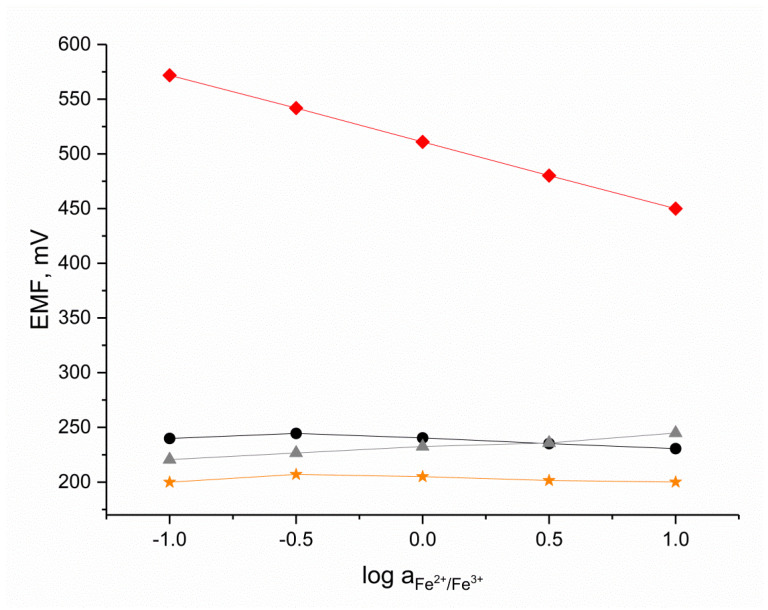
Redox sensitivity test performed for 🟊 planar H^+^-selective electrode, ▲ coated-disc H^+^-selective electrode, ⏺ glass electrode, and ◆ platinum electrode in iron chloride solution with log of Fe^2+^/Fe^3+^ ratio equal to −1, −0.5, 0, 0.5, and 1.

The redox sensitivity test was conducted for the planar electrode with the hydrogen-selective membrane, as well as for the coated-disc electrode, the glass electrode, and platinum electrode, which was used as a control electrode. The potentiometric response was recorded in solutions containing a constant amount of a FeCl_2_ and FeCl_3_ redox couple (1 mM) with a logarithm of Fe^2+^/Fe^3+^ ratio equal to −1, −0.5, 0, 0.5, and 1. The obtained results are presented in Figure 6.

A slight change in the potential was caused by various pH values in the examined solutions than by the redox signal; therefore, there was no redox response detected in the H^+^- selective electrodes, both in the case of the planar and coated-disc electrode. The test proved that the examined pH sensors are not redox-sensitive.

The platinum electrode exhibited a near-Nernstian response with a change in the log Fe^2+^/Fe^3+^ value equal to 60 mV/dec, as expected.

### 3.7. pH Sensitivity Test

**Figure 7:**
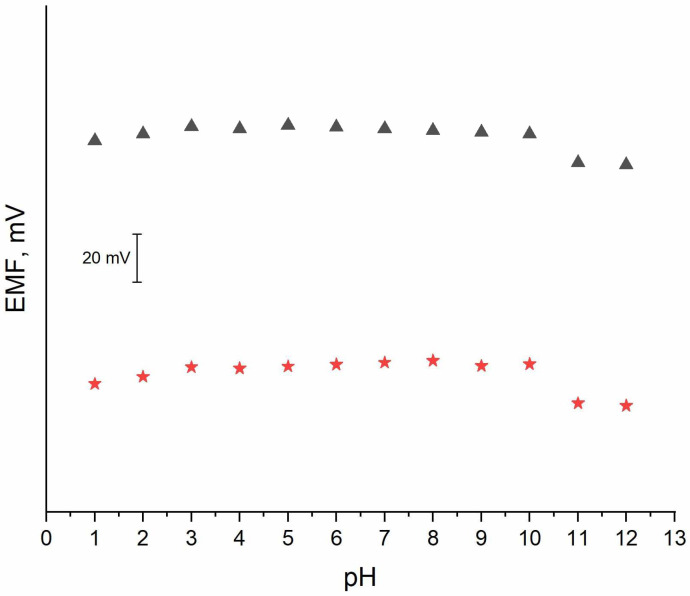
pH sensitivity test performed for 🟊 planar K^+^-selective electrode and ▲ coated-disc K^+^-selective electrode in the standard K^+^ ion solution with pH adjusted from 1 to 12.

The pH sensitivity test was conducted for the planar electrode with the potassium-selective membrane, as well as for the coated-disc electrode. The influence of the pH value on the potentiometric response was tested in the 10^−1^ M solution of potassium ions. The pH values of the KCl solutions were adjusted with the use of concentrated HCl or NaOH solutions. The standard K^+^ ion solution was titrated with sodium hydroxide to obtain solutions with a pH ranging from 6 to 12, and hydrochloric acid was used to obtain lower pH values (2–5). As can be seen in Figure 7, a stable EMF value was observed for pH values of 3 to 10. The test has shown that the planar K^+^-selective electrode presented in this work is not susceptible to changes in pH values between pH 3 and 10.

### 3.8. Resistance and Capacitance of Sensors

#### 3.8.1. EIS

The electrochemical impedance spectroscopy (EIS) method was applied to evaluate the electrical properties of planar electrodes, such as capacitance and the resistance parameter.

**Figure 8:**
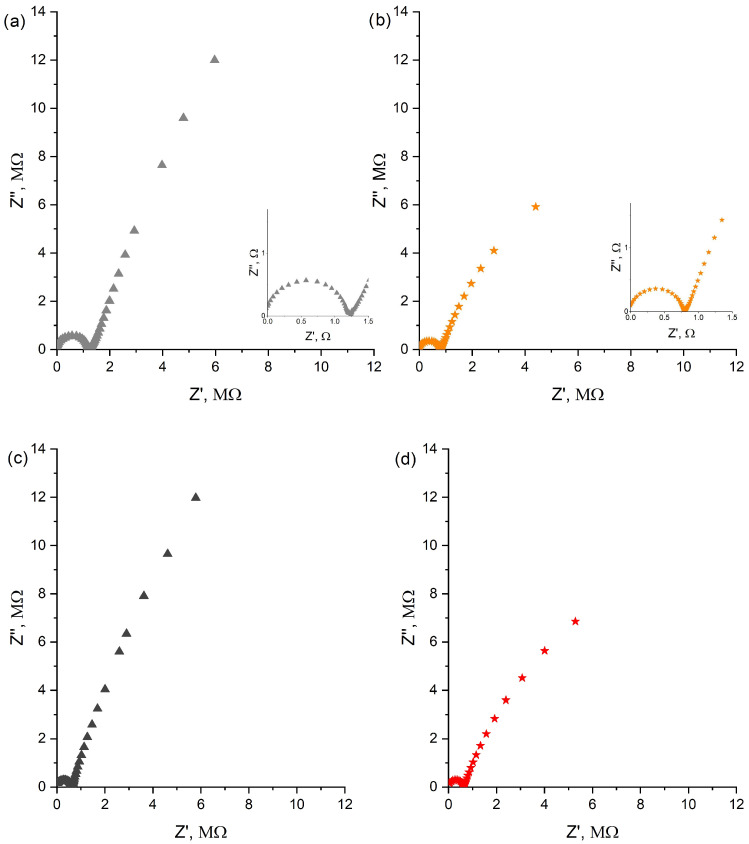
Nyquist plots recorded in standard solution of pH 3 for ▲ coated-disc H^+^-selective electrode (**a**) and 🟊 planar H^+^-selective electrode (**b**); K^+^ ion standard solution of 10^−1^ M concentration for ▲ coated-disc K^+^-selective electrode (**c**) and 🟊 planar K^+^-selective electrode (**d**). Insets to subfigures (**a**,**b**) present enlarged semicircles obtained for each electrode used to determine resistance parameter and equivalent circuit used to evaluate all tested electrodes.

The resistance parameter can be read as the diameter of the semicircle on the Nyquist plot (Figure 8) in the high-frequency region and evaluated based on the equivalent circuit fitted to the results of the electrochemical impedance spectroscopy.

The EIS spectra were fitted to the electrical circuit in which the Constant Phase Element (CPE) was used instead of the capacitor. On the basis of the charge-transfer resistance (R_ct_), the Warburg coefficient (W), and differential capacity (C_dl_) of the electrical double layer, the electrical parameters were calculated. The equivalent circuit is provided in the inset of Figure 8. The same circuit was used to evaluate the electrical properties of all tested electrodes.

Based on the data provided by the fitting software (Nova 2.1 by Metrohm Autolab, Utrecht, The Netherlands), the resistance of the pH planar electrode is 0.83 MΩ, which is lower compared to the resistance of the coated-disc electrode (1.28 MΩ).

This suggests that the ion-to-electron transduction processes are more efficient in planar electrodes with two layers of membranes.

For the planar potassium-selective electrode, the resistance parameter obtained based on the equivalent circuit equals 0.61 MΩ.

The electrical capacitance parameter can be calculated based on the impedance recorded for the lowest frequency using C = C = 1/(2πƒZ^″^). The capacitance parameter of the planar pH electrode is equal to 2.69 µF, which is higher compared to the capacitance of the coated-disc electrode (1.32 µF). Also, for the planar potassium-selective electrode, the electrical capacitance parameter turned out to be almost twice as high as for the coated-disc potassium-selective electrode.

The resistance and capacitance parameters obtained from all electrodes tested were collected and are compared in Table 2.

#### 3.8.2. Chronopotentiometry

The chronopotentiometry method was incorporated to evaluate the electrical properties of planar sensors. Chronopotentiograms (potential response of the electrode (E_dc_) with time (t)) were recorded while applying the current of +1 nA for 60 s and, alternately, −1 nA for another 60 s. This procedure was repeated three times for each electrode.

Resistance (R) is calculated based on the potential jump value (△E_dc_) using the R = △E_dc_/2I equation. This method also confirmed that the application of an additional membrane lowers the resistance value from 2.0 MΩ (resistance of the H^+^-selective coated-disc electrode) to 1.2 MΩ (resistance of the planar pH electrode).

The electrical capacitance parameter was calculated using the equation ΔE_dc_/Δt = I/C, where ΔE_dc_/Δt is the potential drift and I stands for the current forced to flow through the cell. As it was evaluated using EIS, the electrical capacitance of the planar electrodes is twice that of the coated-disc electrodes.

The electrical parameters of K^+^ and H^+^-selective sensors with one membrane and two membranes obtained using electrochemical impedance spectroscopy and chronopotentiometry are compared in Table 2.

### 3.9. In Situ Analysis of Real Samples

The application of a designed planar sensor was examined during the in situ measurement conducted in the flowerpot with a plant (orchid). Both the planar and coated-disc electrode were placed into the soil directly in the pot to evaluate the concentrations of hydrogen and potassium ions. The measurement was carried out by introducing the sensor into the soil, and the EMF was recorded to evaluate the concentrations of ions in the soil. Furthermore, the influence of the fertilization process on the concentrations of potassium and hydrogen ions was tested. The results are presented in Table 3.

The obtained results confirm that the designed planar electrode is a reliable and convenient tool for in situ measurements in environmental samples. The standard deviation values, indicating the repeatability of the sensor, are lower for the planar sensor, but it is also much easier to conduct the measurement using this construction of an electrode. The coated-disc electrode, with a membrane placed on the surface that is pressed into the soil, is destroyed easily because the membrane tends to peel off an electrode. The planar construction with two membranes on the side surface of a sensor is more durable and resistant to the measurement environment. Furthermore, the sharp end of the body of an electrode makes it easier to place the electrode in the sample.

By changing the sensor’s construction, it is possible to obtain a more precise and durable analytical tool compared to the well-known coated-disc electrode without the need to apply additional materials or layers.

## 4. Discussion

The results obtained for the planar sensor were compared with the results presented so far in the literature for various constructions of sensors. The parameters were studied for both hydrogen- (Table 4) and potassium-selective (Table 5) ISEs. The tables allow for a comparison of the analytical parameters of sensors, such as the slope and linear range of the calibration curve and the potential stability and electrical parameter, which is the electrical capacity. The electrical capacity parameter was compared based on the results obtained using the chronopotentiometry method.

Based on the results collected within Table 4, it can be concluded that the planar pH sensor presented in the scope of this work exhibits competitive analytical performance compared to the other solutions presented so far in the literature. In comparison with multiwalled carbon nanotubes and iridium dioxide and ruthenium dioxide-based SC-ISEs, the planar double-membrane sensor exhibits better potential stability during the course of the measurement despite the lack of an extra material layer. However, the high electrical capacity of iridium dioxide–carbon nanotubes and iridium dioxide–carbon nanotubes–poly(3-octylthiophene)-based SC-ISEs ensured enhanced potential stability of only 0.077 and 0.036 mV/h, respectively.

In contrast to the screen-printed electrodes with ruthenium dioxide and ruthenium dioxide hydrate, the planar sensor turned out to exhibit a wider linear range. In terms of the potentiometric response, the widest pH range can be attributed to the SC-ISEs with a ruthenium dioxide layer (2–12); however, without the application of an additional material layer, we were able to obtain a range that was almost as wide with pH 2–11.

By taking into consideration all of the collected data, it can be concluded that despite the electrical capacity being much lower in comparison to that of other sensors, the potassium-selective planar sensor exhibits analytical parameters that are nearly as great. The potential drift is more significant in contrast to SC-ISEs; however, the linear range is competitive and is one of the widest among those of other sensors.

The planar double-membrane sensor construction should be compared with the double-membrane construction in which two membranes are placed opposite to each other. For the planar construction, we were able to obtain a lower detection limit.

Based on the collected data for the pH and potassium-selective SC-ISEs, it can be concluded that despite the lack of an additional material in the sensor’s construction, we were able to obtain a potentiometric sensor with analytical properties competitive to those of other solutions that are usually more advanced and expensive.

The screen-printed and double-membrane electrodes turned out to exhibit narrower linear ranges in comparison with our planar H^+^ and K^+−^ selective sensors.

## 5. Conclusions

The new construction of an ion-selective electrode presented in the scope of this paper exhibits enhanced analytical, electrical, and performance parameters in reference to the well-known coated-disc electrode with one membrane. This type of sensor can be particularly useful for in-situ measurements in environmental samples. The unique shape and construction ensure the ease of measurement and durability of the sensor.

The planar construction of an electrode was tested on the example of potassium- and hydrogen-selective sensors. The conducted test confirmed that this construction idea is universal and can be applied to the design of sensors selective to various ions. The presence of an additional membrane on the opposite side of the sensor allowed for the electrical properties of the sensors to be enhanced. The obtained results suggest that the ion-to-electron transduction processes are more efficient in the planar electrodes with two layers of membranes. For the planar electrodes, a higher electrical capacity and lower resistance were observed due to the higher efficiency of the charge transfer processes.

As a consequence, for both tested membranes, the analytical properties of the planar electrode turned out to be improved in contrast to those of the coated-disc electrode. For the K^+^ and H^+^-selective planar sensors, wider linear ranges, an enhanced potential stability, and a shorter response time were observed. The potentiometric response of planar electrodes is more repeatable, reversible, and more stable compared to that of the coated-disc electrode with one membrane. Additionally, the pH planar sensor can be considered as an alternative for a glass electrode in the pH range between 2 and 11. The designed planar pH sensors turned out to be redox-insensitive, and planar potassium sensors do not exhibit sensitivity toward H^+^ ions in the pH range of 3 to 10.

Moreover, the preparation method of the sensor is as easy as possible, and the planar construction ensures a smaller size, durability, and resistance to mechanical damage. What should be emphasized is that this construction idea allows the sensor properties to be improved without using any additional materials, such as layers or additives, in the membrane. The practical application of the construction of a new sensor was examined during the in situ measurement in the soil.

## Figures and Tables

**Table 1:** Analytical parameters include slope, standard potential, and linear range of selective H^+^ and K^+^ electrodes with standard deviation values that describe repeatability. Values were obtained based on 5 calibrations performed over one week (n = 5).

Ion, X	Electrode	Slope S ± SD [mV/pX]	Standard Potential E^0^ [mV]	Linear Range [M]
H^+^	planar electrode	56.6 ± 0.2	366 ± 3	5 × 10^−11^–10^−2^
	coated-disc electrode	56.6 ± 4.5	384 ± 14	5 × 10^−10^–10^−2^
	commercial glass electrode	59.0 ± 0.1	415 ± 2	10^−12^–10^−2^
K^+^	planar electrode	55.2 ± 0.2	375 ± 3	10^−6^–10^−1^
	coated-disc electrode	55.1 ± 4.5	480 ± 12	10^−5^–10^−1^

**Table 2:** Electrical parameters of electrodes with standard deviation values (n = 5) evaluated using electrochemical impedance spectroscopy and chronopotentiometry method.

Ion	Electrode	Electrochemical Impedance Spectroscopy		Chronopotentiometry	
		Resistance ± SD [kΩ]	Capacitance ± SD [µF]	Resistance ± SD [kΩ]	Capacitance ± SD [µF]
H^+^	planar electrode	775 ± 5	2.69 ± 0.08	1161 ± 11	2.72 ± 0.04
	coated-disc electrode	1292 ± 9	1.32 ± 0.07	1922 ± 19	1.30 ± 0.04
K^+^	planar electrode	614 ± 4	2.32 ± 0.06	762 ± 9	2.65 ± 0.04
	coated-disc electrode	624 ± 8	1.33 ± 0.08	1367 ± 19	0.95 ± 0.03

**Table 3:** Concentrations of hydrogen and potassium ions in flowerpot’s soil with standard deviation values (n = 5) evaluated using planar and coated-disc electrodes during in situ measurement (* result obtained for two different electrodes due to damage to ion-selective membrane).

Ion	Electrode	Ion Concentration during In situ Measurement in Flowerpot with Plant	
		Before Fertilization	After Fertilization
H^+^ (pH)	coated-disc electrode	7.86 ± 0.11 *	6.05 ± 0.12
	planar electrode	7.86 ± 0.06	6.07 ± 0.04
K^+^ (pK)	coated-disc electrode	2.95 ± 0.08 *	2.12 ± 0.06 *
	planar electrode	2.95 ± 0.03	2.12 ± 0.03

**Table 4:** The comparison of electrical and analytical parameters of ion-selective electrodes sensitive to hydrogen ions (pH).

Sensor	Slope [mV/pH]	Linear Range	Electrical Capacity [μF]	Potential Drift [mV/h]	Reference
SC-ISE with multi-walled carbon nanotube layer	58.8	2.89–9.90	30	0.5	[25]
SC-ISE with carbon nano-onions poly(dopamine) layer	60.1	1.50–10.50	-	-	[26]
SC-ISE with poly(aniline) layer	52.7	2.0–9.0	-	-	[27]
SC-ISE with ruthenium dioxide layer	59.31	2.0–12.0	1120	0.15	[28]
SC-ISE with iridium dioxide layer	54.12	2.0–11.0	66	0.1	[29]
SC-ISE with iridium dioxide-carbon nanotubes layer	54.40	2.0–11.0	174	0.077	[29]
SC-ISE with iridium dioxide-carbon nanotubes- poly(3-octylthiophene) layer	57.18	2.0–11.5	387	0.036	[29]
Screen-printed ruthenium dioxide ISE	51.20	2.0–8.0	-	-	[17]
Screen-printed ruthenium dioxide hydrate ISE	52.11	2.0–10.0	-	-	[18]
Planar double-membrane sensor	56.56	2.0–11.0	2.72	0.12	this work

**Table 5:** Comparison of electrical and analytical parameters of ion-selective electrodes sensitive to potassium ions.

Sensor	Slope [mV/pK]	Linear Range [M]	Electrical Capacity [μF]	Potential Drift [mV/h]	Reference
SC-ISE with 7,7,8,8-tetracyanoquinodimethane layer	58.68	10^−1^–10^−6.5^	132	0.011	[30]
SC-ISE with ordered mesoporous carbon layer	63.5	10^−0.2^–10^−4.2^	-	0.028	[31]
SC-ISE with platinum nanoparticles layer	59.51	-	82	0.028	[32]
SC-ISE with graphene layer	59.2	10^−1^–10^−4.5^	91	-	[33]
SC-ISE with carbon black layer	59.0	10^−1^–10^−6.5^	1391	0.001	[34]
SC-ISE with molybdenum dioxide layer	55.0	10^−3^–10^−5^	86	0.012	[35]
SC-ISE with manganese dioxide layer	51.85	10^−2^–10^−5^	29	-	[36]
SC-ISE with copper oxide layer	56.68	10^−1^–10^−5^	0.104	0.54	[37]
SC-ISE with ruthenium dioxide layer	57.37	10^−1^–10^−6^	1233	0.0015	[38]
SC-ISE with iridium dioxide layer	59.29	10^−6–^10^−1^	920	0.063	[39]
Double-membrane ISE	55.50	1.0–10^−4^	-	0.033	[14]
Planar double-membrane sensor	55.20	10^−1^–10^−6^	2.65	0.092	this work

## Data Availability

Data are contained within the article.
